# GLP-1 Receptor Agonist Effects on Lipid and Liver Profiles in Patients with Nonalcoholic Fatty Liver Disease: Systematic Review and Meta-Analysis

**DOI:** 10.1155/2021/8936865

**Published:** 2021-11-12

**Authors:** Shahla Rezaei, Reza Tabrizi, Peyman Nowrouzi-Sohrabi, Mohammad Jalali, Stephen L. Atkin, Khalid Al-Rasadi, Tannaz Jamialahmadi, Amirhossein Sahebkar

**Affiliations:** ^1^Student Research Committee, Shiraz University of Medical Sciences, Shiraz, Iran; ^2^Department of Clinical Nutrition, School of Health & Nutrition, Shiraz University of Medical Sciences, Shiraz, Iran; ^3^Nutrition Research Center, School of Nutrition and Food Sciences, Shiraz University of Medical Sciences, Shiraz, Iran; ^4^Noncommunicable Diseases Research Center, Fasa University of Medical Sciences, Fasa, Iran; ^5^Department of Biochemistry, School of Medicine, Shiraz University of Medical Sciences, Shiraz, Iran; ^6^Weill Cornell Medicine Qatar, Doha, Qatar; ^7^Medical Research Centre, Sultan Qaboos University, Muscat, Oman; ^8^Department of Nutrition, Faculty of Medicine, Mashhad University of Medical Sciences, Mashhad, Iran; ^9^Applied Biomedical Research Center, Mashhad University of Medical Sciences, Mashhad, Iran; ^10^Biotechnology Research Center, Pharmaceutical Technology Institute, Mashhad University of Medical Sciences, Mashhad, Iran; ^11^School of Pharmacy, Mashhad University of Medical Sciences, Mashhad, Iran

## Abstract

**Aims:**

This meta-analysis of randomized placebo-controlled clinical trials assessed the effect of glucose-like peptide-1-receptor agonists (GLP-1RA) on the lipid profile and liver enzymes in patients with nonalcoholic fatty liver disease (NAFLD).

**Materials and Methods:**

Randomized placebo-controlled trials investigating GLP-1RA on the lipid profile and liver enzymes in patients with NAFLD were searched in PubMed-Medline, Scopus, Web of Science, and Google Scholar databases (from inception to January 2020). A random-effects model and a generic inverse variance method were used for quantitative data synthesis. Sensitivity analysis was conducted. Weighted random-effects meta-regression was performed on potential confounders on lipid profile and liver enzyme concentrations.

**Results:**

12 studies were identified (12 GLP-1RA arms; 677 subjects) that showed treatment with GLP-1RA reduced alanine transaminase (ALT) concentrations (WMD = −10.14, 95%CI = [−15.84, −0.44], *P* < 0.001), gamma-glutamyl transferase (GGT) (WMD = −11.53, 95%CI = [−15.21,−7.85], *P* < 0.001), and alaline phosphatase (ALP) (WMD = −8.29, 95%CI = [−11.34, −5.24], *P* < 0.001). Aspartate aminotransferase (AST) (WMD = −2.95, 95% CI = [−7.26, 1.37], *P*=0.18) was unchanged. GLP-1 therapy did not alter triglycerides (TC) (WMD = −7.07, 95%CI = [−17.51, 3.37], *P*=0.18), total cholesterol (TC) (WMD = −1.17 (−5.25, 2.91), *P*=0.57), high-density lipoprotein (HDL-C) (WMD = 0.97, 95%CI = [−1.63, 3.58], *P*=0.46), or low-density lipoprotein (LDL-C) (WMD = −1.67, 95%CI = [−10.08, 6.74], *P*=0.69) in comparison with controls.

**Conclusion:**

The results of this meta-analysis suggest that GLP-1RA treatment significantly reduces liver enzymes in patients with NAFLD, but the lipid profile is unaffected.

## 1. Introduction

Nonalcoholic fatty liver disease (NAFLD) is an increasing global public health problem with a worldwide prevalence of NAFLD estimated at approximately 25% [1] and a common cause of chronic liver disease [2], and it is predicted to develop in more than 30% of the US adult population [3]. NAFLD is diagnosed when there is hepatic steatosis in the absence of other causes of hepatic fat [4]. In NAFLD, there is an accumulation of fat in the liver through increased free fatty acid delivery to the liver, increasing triglyceride synthesis, decreasing triglyceride export, and reducing beta-oxidation [5]. Coexisting insulin resistance (IR) in NAFLD enhances lipolysis from the adipose tissue [5]. Currently, there are no approved drug treatments for NAFLD and NASH [6].

Glucagon-like peptide-1 receptor agonists (GLP-1RA) are a newly introduced class of antidiabetic drugs that improve glycemic control via several molecular pathways [7, 8]. These pharmacologic agents reduce blood glucose via glucose-dependent insulin secretion and by glucagon suppression [8]. In addition, GLP-1RAs have other beneficial effects [9–16] and decrease energy intake and body weight by prolonging gastric emptying and inducing satiety [17]. There is an association between NAFLD and metabolic syndrome that causes DM, dyslipidemia, and obesity suggesting that breaking this cycle by GLP-1 agonists may have therapeutic potential [18], particularly as they may have anti-inflammation activity [19]. The administration of the GLP-1RA liraglutide was suggested to directly reduce liver fibrosis and steatosis in an in vivo study [17] and reduces markers of fibrosis in man [20]. Therefore, GLP-1 receptor analogue therapy may have the potential for the treatment of NAFLD and NASH patients; however, it is unclear from the studies that have been done whether GLP-1 agonists improve the hepatic enzyme and lipid profiles in subjects with NAFLD; therefore, this systematic review and meta-analysis were undertaken.

## 2. Methods

### 2.1. Search Strategy

This meta-analysis was conducted according to PRISMA instruction of systematic reviews and meta-analysis [21]. The scientific web-portals such as PubMed, Scopus, Cochrane, Web of Science, Embase, and Scholar were carefully surveyed to extract all relevant literature on the effects of GLP-1 receptor agonists on lipid profile and liver enzymes in patients with nonalcoholic fatty liver disease published until January 2020. The key terms that were applied to finalize the first step of the search strategy to gather target data are shown in Appendix. Additionally, manual searches were performed to find articles that were not indexed in target databases. Only human-based studies were selected from the search strategy, and language restriction was not considered. Two authors (Sh.R. and P.N.) independently surveyed the title and abstracts of the classified papers, extracted relevant data, and applied quality assessments of eligible studies. A third author (R.T.) checked the data and resolved all disagreements.

### 2.2. Study Selection

The following strategy was utilized to select target papers: randomized clinical trials (parallel or cross-over) that investigated the effect of GLP-1 receptor agonists on the lipid profile and liver enzymes in patients with nonalcoholic fatty liver disease, individuals treated with GLP-1 receptor agonists that were compared with placebo-control or other pharmaceutical agents, at least 12 weeks' administration of GLP-1 receptor agonists, papers that contained data for standard deviation (SD), standard error (SE), and confidence interval (CI) parameters in the beginning and the end of each study for both the intervention and control groups.

### 2.3. Data Extraction

Relevant RCT data were extracted by rechecking the name of first author, country, the number of individuals in the intervention and control groups, the type and doses of GLP-1 receptor agonists, duration of the study, type of the study, and related data for analysis (Table 1). For each study, the values of the mean and SD for lipid profile and liver enzymes were recorded at the beginning and the end of each study using the calculation of the difference between the values before and after the intervention. The following formula was used to calculate the mean difference of SDs:

(1) $$\begin{array}{c} \text{SD}=\text{square root}\left[{\left(\text{SD baseline}\right)}^{2}+{\left(\text{SD end of study}\right)}^{2}-\left(2r\times \text{SD baseline}\times \text{SD end of study}\right)\right]. \end{array}$$

A correlation coefficient of 0.5 was used for *r*, estimated between 0 and 1 values [22]. The formula $\text{SD}=\text{SE}\times \sqrt{n}$ (*n* = the number of individuals in each group) was used to measure SD in each article that reported SE instead of SD.

### 2.4. Quality Assessment

**Figure 1:**
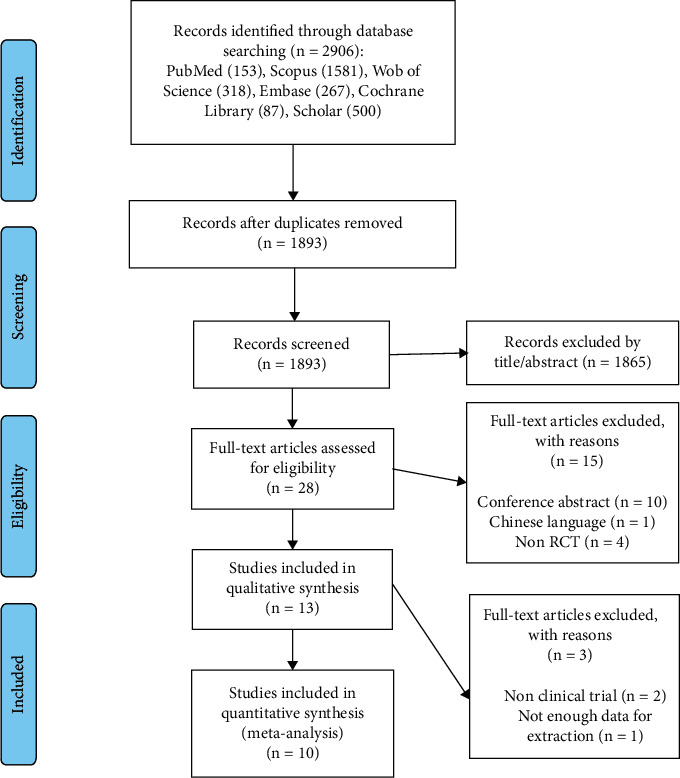
Flowchart of study selection method.

The quality assessment of the included papers in this meta-analysis was conducted based on Cochrane criteria [23]. Accordingly, any source of bias, including selection bias, performance bias, detection bias, attrition bias, and reporting bias, was judged for all included studies (Figure 1).

### 2.5. Statistical Analysis

A random-effects model was performed using Stata v.13 (StataCorp. 2021, Stata Statistical Software: release 17; College Station, TX: StataCorp. LLC) to obtain weighted mean difference (WMD) and corresponding 95% CIs. Interstudy heterogeneity was investigated by checking Cochrane's Q test (*I*^2^ > 50%, *P* < 0.1) [24]. In cases with a high amount of statistical heterogeneity, a random-effects meta-regression was applied to find its potential source by confounders such as age, intervention duration, baseline body weight, and body mass index (BMI). Subgroup calculation was conducted according to the age (≥50 years, <50 years), study duration (≤12 weeks vs >12 weeks), BMI (>30, <30), body weight (>85 kg, <85 kg), and type of intervention (GLP-1 vs. GLP-1 plus other treatment) to detect the source of heterogeneity. Overall sensitivity analysis was performed to assess the dependence of pooled results by discarding each study in turn.

Estimation of the value of a correlation coefficient (*r*) in each outcome was imputed from studies that reported the SD of change for each intervention group in the current meta-analysis. The following formula was used to determine the SD of change calculation among studies that did not provide sufficient information [24]:

(2) $$\begin{array}{c} R=\left[\frac{R=\left({\text{SD}}_{\text{pre}}^{2}+{\text{SD}}_{\text{post}}^{2}-{\text{SD}}_{\text{Change}}^{2}\right)}{2\times {\text{SD}}_{\text{pre}}\times {\text{SD}}_{\text{post}}}\right], \end{array}$$

where *R* was for TC: 0.81, TG: 0.45, HDL-c: 0.50, LDL-C: 0.68, AST: 0.64, Alt: 0.62, GGT: 0.60, and ALP: 0.50. We also conducted a sensitivity analysis for outcomes (TG, AST, and ALT) with different values of *r*; TG (0.26 and 0.63), AST (0.20 and 0.77), and ALT (0.40 and 0.82) to evaluate if the pooled results are sensitive to these levels.

### 2.6. The Grade Profile

The overall evaluation of the evidence relating to the outcomes was conducted by the Grading of Recommendations Assessment, Development, and Evaluation (GRADE) approach (Table 2) [25].

## 3. Results

### 3.1. Search Results and Study Selection

The flowchart explaining the method of selection and references obtained in the databases is shown in Figure 1. In total, 2906 articles were identified in the first phase of the literature search. After removal of duplicate studies (*n* = 1013), irrelevant studies according to the title and abstracts (*n* = 1865), different type of intervention (*n* = 4), conference abstracts (*n* = 10), and Chinese language (*n* = 1), thirteen potentially eligible studies were considered for full-text review. Subsequently, three articles were excluded for the following reasons: type of study and insufficient data reporting outcomes. Ultimately, ten studies were entered in the current meta-analysis.

### 3.2. Data Charachteristics

**Figure 2:**
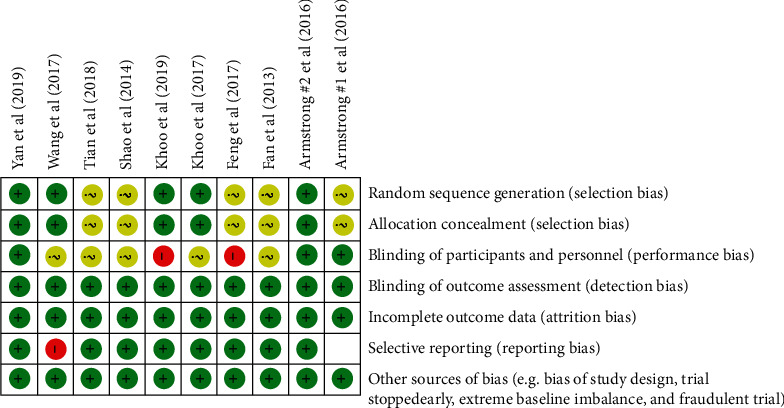
Details of quality assessment of the included papers.

The main characteristics of the included trials are shown in Table 1. All of the RCTs were published between 2013 and 2019, were conducted in China [26–31], Singapore [32, 33], and UK [34, 35], and lasted 12 to 48 weeks. A total of 677 participants were aged between 18 to 70 years. Seven studies used Liraglutide as an intervention [28, 30–35], and two others [26, 27, 29] used exenatide plus other treatments. The details of the quality assessment are shown in Figure 2.

### 3.3. The Effects of GLP-1 Receptor Agonists on Lipid Profile

**Figure 3:**
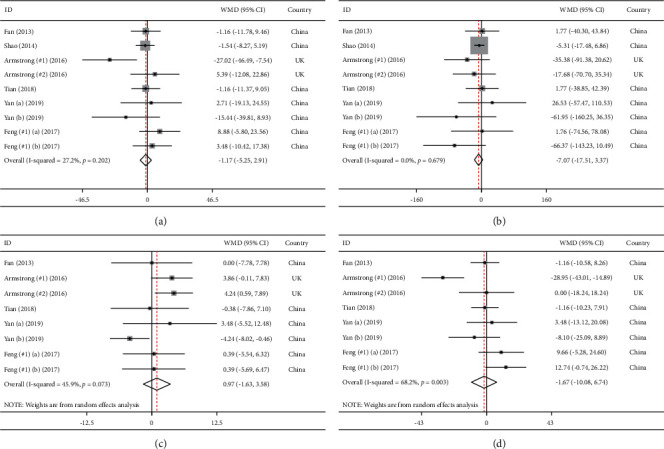
Meta-analysis of weighted mean differences estimates for lipid profiles including (a) total cholesterol, (b) triglycerides, (c) HDL-cholesterol, and (d) LDL-cholesterol in intervention and placebo groups (CI = 95%).

The results of the meta-analysis regarding the influence of GLP-1 receptor agonists are shown in Figure 3. Pooled effect sizes indicated that receiving GLP-1 receptor agonists did not cause a statistically significant change in serum TG (WMD = −7.07, 95% CI = [−17.51, 3.37], *P*=0.18), TC (WMD = −1.17, 95% CI = [−5.25, 2.91], *P*=0.57), HDL-C (WMD = 0.97, 95% CI = [−1.63, 3.58], *P*=0.46), and LDL-C (WMD = −1.67, 95% CI = [−10.08, 6.74], *P*=0.69) in comparison with controls.

In addition, based on Cochrane's Q test, low degree of between-study heterogeneity was observed in TG (*I*^2^ = 0.0%, *P*=0.6), TC (*I*^2^ = 27.2%, *P*=0.2), and HDL-C (*I*^2^ = 45.9%, *P* < 0.1). Conversely, LDL-C (*I*^2^ = 68.2%, *P* < 0.1) had a high amount of statistical heterogeneity.

### 3.4. The Effects of GLP-1 Receptor Agonists on Liver Enzymes

**Figure 4:**
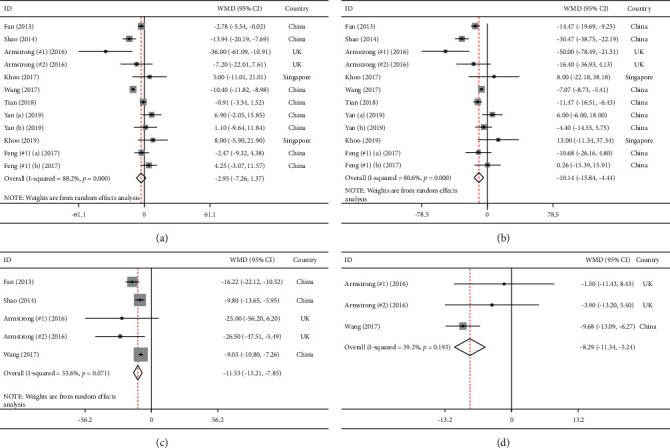
Meta-analysis of weighted mean differences estimates for liver enzymes including (a) aspartate aminotransferase, (b) alanine aminotransferase, (c) gamma-glutamyltransferase, and (d) alkaline phosphatase in intervention and placebo groups (CI = 95%).

Figure 4 presents the results of meta-analysis for liver enzymes. Treatment with GLP-1 receptor agonists lead to the amelioration of ALT serum concentration (WMD = −10.14, 95% CI = [−15.84, −4.44], *P* < 0.001), GGT (WMD = −11.53, 95% CI = [−15.21, −7.85], *P* < 0.001), and ALP (WMD = −8.29, 95% CI = [−11.34, −5.24], *P* < 0.001). However, serum AST level (WMD = −2.95, 95% CI = [−7.26, 1.37], *P*=0.18) was not significantly affected following intervention.

Regarding between-study heterogeneity, Cochrane's Q test showed the following results: ALT (*I*^2^ = 80.6%, *P* < 0.1), AST (*I*^2^ = 88.2%, *P* < 0.1), ALP (*I*^2^ = 39.2%, *P*=0.19) and GGT (*I*^2^ = 53.6%, *P* < 0.1).

### 3.5. Subgroup Analysis

As shown in Table 3, lipid profiles were not changed based on subgroup analysis. Conversely, AST and ALT were significantly affected when we conducted a subanalysis on duration (≤12 weeks). However, serum ALT was significantly changed when subjects received GLP-1 agonists alone, and serum AST was reduced when they received another treatment along with GLP-1 agonists. In addition, the AST level was altered in older participants (≥50 years).

### 3.6. Sensitivity Analysis and Publication Bias

The sensitivity analysis was applied using “one-study-removed” strategy to investigate the influence of each study on the effect size. The results of sensitivity analysis displayed that the pooled results of interested outcomes were not sensitive to each study. Additionaly, we checked triglycerides, aspartate aminotransferase, alanine aminotransferase (TG, AST, and ALT) with different values of *r*. The significance of the results of TG (*r* = 0.26: *I*^2^ = 0.0%, *P*=0.22; *r* = 0.63: *I*^2^ = 0.0%, *P*=0.13), AST (*r* = 0.20: *I*^2^ = 83.7%, *P*=0.18; *r* = 0.77: *I*^2^ = 99.4%, *P*=0.05), and ALT (*r* = 0.40: *I*^2^ = 76.3%, *P* < 0.001; *r* = 0.82: *I*^2^ = 86.5%, *P*=0.001) was independent of different values of *r*. Due to the minimum number of studies required for the assessment of publication bias by funnel plot being 10 and for Egger's test being 20, these tools for detection of publication bias would not be meaningful with so few studies and therefore were not performed.

## 4. Discussion

This meta-analysis showed that the combined available studies, including liraglutide and exenatide, showed an improvement in the liver enzymes of patients with NAFLD but that the lipid profile was unchanged. This suggests that GLP-1 agonists may have utility in the treatment of NAFLD or at least prevention of further progression. Similarly, hepatic histological features in patients with nonalcoholic steatohepatitis (NAFLD with additional inflammation) were improved in the liraglutide group compared to placebo (hepatocyte ballooning (61% vs. 32%) and steatosis (83% vs. 45%) [34]. Moreover, in a recent meta-analysis of four clinical trials, histological improvement was demonstrated [36]. The mechanism by which liraglutide may improve NAFLD could be through inhibiting the NLRP3 inflammasome and pyroptosis activation through mitophagy [37]. Currently, there is no recognized therapeutic agent for the treatment of NAFLD [38]; however, whilst the studies with these GLP-1 agonists may be encouraging, they are of too short a study duration to know if their effects are maintained or that they have continued clinical therapeutic utility.

Liraglutide is reported to have a cholesterol-lowering effect though the mechanism is unclear [39], and others have shown an improvement in lipids in nondiabetic subjects [20, 40]. In this meta-analysis, there was no effect of GLP-1 agonists on any of the lipid parameters, including TG, TC, HDL-C (where the heterogeneity between studies was low), and LDL-C (where the heterogeneity between studies was high). This suggests that GLP-1 agonists do not have a direct effect on lipid metabolism in NAFLD and that the lipid changes reported in the literature may have been indirectly due to associated weight loss through the satiety effects of the GLP-1 agonists such as liraglutide [17].

The strength of this study was that it focused on randomized clinical trials that would increase its power. This meta-analysis has a number of limitations. Firstly, the effects of GLP-1 therapy on liver enzymes and the lipid profile in NAFLD were not the primary aim of the clinical trials and the studies were not powered for this. Secondly, there were only 12 trials with relatively few subjects available to be analyzed, giving a modest though robust number of subjects to undertake the analysis. The meta-analysis was also limited in that only two studies were with exenatide and the remainder was with liraglutide and no studies were available for the newer GLP-1 agonists such as semaglutide. Since GLP-1 agonists have differing structures and potencies, their effects on liver enzymes are also likely to be different [41].

## 5. Conclusion

The results of this meta-analysis suggest that GLP-1 agonist treatment significantly reduces the liver enzymes ALT, GGT, and ALP, though AST was no different in patients with NAFLD; however, the lipid profile is unaffected.

## Figures and Tables

**Table 1:** Characteristics of included studies.

First author	Publication year	Country	Population	Age (control vs. intervention)	Sample size (control vs. intervention)	Type of study	Type of intervention	Control group	Duration
Fan et al.	2013	China	NAFLD + T2DM + obesity	54.68 ± 12.14; 51.02 ± 10.10	68/49	RCT	Exenatide	Metformin	12 weeks

Shao et al.	2014	China	NAFLD + T2DM	42 ± 3.2; 43 ± 4.1	30/30	RCT	Exenatide + insulin glargine	Insulin aspart + insulin glargine	12 weeks

Armstrong et al.	2016	UK	NASH with/without T2DM	18–70	7/7	Double-blind, randomized, placebo-controlled trial	Liraglutide	Placebo	12 weeks

Armstrong et al.	2016	UK	NASH with/without T2DM	50 ± 12; 50 ± 11	22/23	Multicentre, double-blinded, randomized, placebo-controlled phase 2 trial	Liraglutide	Placebo	48 weeks

Khoo et al.	2017	Singapore	NAFLD + obesity	43.7 ± 10.4; 39.0 ± 8.1	12/12	Pilot randomized trial	Liraglutide	Diet + exercise	26 weeks

Wang et al.	2017	China	NAFLD + T2DM	40–78; 41–75	49/49	RCT	Exenatide + metformin	Metformin	12 weeks

Feng et al.	2017	China	NAFLD + T2DM	48.07 ± 12.59; 46.79 ± 9.68	29/14.5	Single-center, open-label, prospective, and randomized trial with parallel design	Liraglutide	Gliclazide	24 weeks

Feng et al.	2017	China	NAFLD + T2DM	46.31 ± 12.32; 46.79 ± 9.68	29/14.5	Single-center, open-label, prospective, and randomized trial with parallel design	Liraglutide	Metformin	24 weeks

Tian et al.	2018	China	NAFLD + T2DM	56.4 ± 8.4; 58.5 ± 7.6	75/52	RCT	Liraglutide	Metformin	12 weeks

Yan et al.	2019	China	NAFLD + T2DM	45.7 ± 9.2; 43.1 ± 9.7	27/12	Randomized, open-label, active-controlled, parallel-group, multicenter trial	Liraglutide	Sitagliptin	26 weeks

Yan et al.	2019	China	NAFLD + T2DM	45.6 ± 7.6; 43.1 ± 9.7	24/12	Randomized, open-label, active-controlled, parallel-group, multicenter trial	Liraglutide	Insulin glargine + metformin	26 weeks

Khoo et al.	2019	Singapore	NAFLD	43.6 ± 9.9; 38.6 ± 8.2	15/15	Prospective randomized pilot study	Liraglutide	Diet + exercise	26 weeks

**Table 2:** Summary of findings.

Absolute effect WMD (95% CI)	No. of studies	Study design	Risk of bias	Inconsistency	Indirectness	Imprecision	Publication bias	Effect size	GRADE quality
*Effects of GLP-1 on lipid profile*									
TG									
−7.07 [−17.51, 3.37]	9	RCT	−1	−1^¥^	0	−2^†^	0	+1	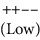
TC									
−1.17 [−5.25, 2.91]	9	RCT	−1^*∗*^	0	0	−1	0	0	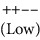
HDL-c									
0.97 [−1.63, 3.58]	8	RCT	−1	0	0	−1^¶^	0	0	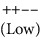
LDL-c									
−1.67 [−10.08, 6.74]	8	RCT	−1	0	0	−2	0	0	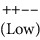

*Effects of GLP-1 on liver enzymes*									
AST									
−2.95 [−7.26, 1.37]	12	RCT	−1	0	0	−2	0	0	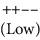 (low)
ALT									
−10.14 [−15.84, −4.44]	12	RCT	−1	0	0	−1	0	+1^‡^	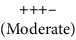 (moderate)

The symbols 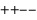 show the quality of evidence. Abbreviations: WMD, weighted mean difference; CI, confidence interval; RCT, randomized controlled trial; TG, triglycerides; TC, total cholesterol; HDL-c, high-density lipoprotein-cholesterol; LDL-c, low-density lipoprotein-cholesterol, AST, aspartate aminotransfrase; ALT, alanine aminotransfrase. ^*∗*^Downgraded one level as the moderate risk of bias. ^¶^Downgraded one level as the confidence interval was moderate. ^†^Downgraded two levels as the number of studies was <5 and imprecision was considerable. ^‡^Upgraded one level due to considerable effect size. ^¥^Downgraded one level as the statistical heterogeneity was >50%.

**Table 3:** The results of subgroup analysis for serum TC, TG, HDL-C, LDL-C, AST, and ALT.

Subgroup		Study	WMD (95% CI)	*P* value	Heterogeneity (*I*^2^)	Meta-regression	Test of group differences *P* > *Q*_*b*
*TG*							
Age	≥50 years old	3	−2.76 (−28.35, 22.83)	0.83	0.0	—	0.718
	<50 years old	6	−7.93 (−19.36, 3.5)	0.17	3.7		
Duration	≤12 weeks	4	−5.47 (−16.48, 5.55)	0.33	0.0	—	0.373
	>12 weeks	5	−21.10 (−17.51, 3.37)	0.20	0.0		
Baseline BMI	>30	4	−7.31 (−18.72, 4.10)	0.21	0.0	—	0.920
	<30	4	−5.86 (−31.57, 19.85)	0.65	0.0		
Baseline body weight	>85 kg	4	−7.31 (−18.72, 4.10)	0.21	0.0	—	0.920
	<85 kg	4	−5.86 (−31.57, 19.85)	0.65	0.0		
Intervention type	GLP-1 agonists	8	−11.96 (−32.24, 8.32)	0.24	0.0	—	0.582
	GLP-1 agonists + other treatment	1	−5.31 (−17.48, 6.86)	0.39	—		

*TC*							
Age	≥50−years old	3	−0.17 (−6.95, 6.61)	0.96	0.0	—	0.719
	<50 years old	6	−1.73 (−6.84, 3.37)	0.50	51.9		
Duration	≤12 weeks	4	−2.93 (−7.74, 1.88)	0.23	52.1	—	0.175
	>12 weeks	5	3.35 (−4.34, 11.05)	0.39	0.0		
Baseline BMI	>30	4	−3.39 (−9.00, 2.21)	0.23	51.0	—	0.256
	<30	4	1.33 (−4.61, 7.28)	0.65	0.0		
Baseline body weight	>85 kg	4	−3.39 (−9.00, 2.21)	0.23	51.0	—	0.256
	<85 kg	4	1.33 (−4.61, 7.28)	0.65	0.0		
Intervention type	GLP-1 agonists	8	−0.95 (−6.08, 4.18)	0.71	36.2	—	0.891
	GLP-1 agonists + other treatment	1	−1.54 (−8.27, 5.19)	—	—		

*HDL-C*							
Age	≥50 years old	3	2.84 (−0.18, 5.86)	0.06	0.0	—	0.307
	<50 years old	5	0.40 (−3.18, 3.98)	0.82	55.8		
Duration	≤12 weeks	3	2.43 (−0.76, 5.63)	0.13	0.0	—	0.460
	>12 weeks	5	0.56 (−3.21, 4.35)	0.76	61.8		
Baseline BMI	>30	4	1.61 (−3.03, 6.26)	0.49	76.1	—	0.619
	<30	4	0.16 (−3.17, 3.50)	0.92	0.0		
Baseline body weight	>85 kg	4	1.61 (−3.03, 6.26)	0.49	76.1	—	0.619
	<85 kg	4	0.16 (−3.17, 3.50)	0.92	0.0		

*LDL-C*							
Age	≥50 years old	3	−1.02 (−7.17, 5.12)	0.74	0.0	0.45 (−1.87, 2.78)	0.890
	<50 years old	5	−2.22 (−18.06, 13.60)	0.78	81.8		
Duration	≤12 weeks	3	−9.48 (−24.70, 5.74)	0.22	83.8	0.36 (−0.37, 1.40)	0.097
	>12 weeks	5	4.79 (−2.48, 12.07)	0.19	5.8		
Baseline BMI	>30	4	−8.96 (−24.42, 6.50)	0.25	71.9	−3.43 (−7.10, 0.22)	0.155
	<30	4	3.26 (−3.46, 10.00)	0.34	30.3		
Baseline body weight	>85 kg	4	−8.96 (−24.42, 6.50)	0.25	71.9	−0.59 (−1.36, 0.18)	
	<85 kg	4	3.26 (−3.46, 10.00)	0.34	30.3		

*AST*							
Age	≥50 years old	4	−5.04 (−11.24, 1.15)	0.11	95.2	−0.31 (−1.22, 0.58)	0.663
	<50 years old	8	−1.60 (−8.97, 5.77)	0.67	80.4		
Duration	≤12 weeks	5	−8.07 (−13.86, −2.29)	0.006	94.4	0.36 (−0.22, 0.95)	0.031
	>12 weeks	7	1.84 (−1.59, 5.28)	0.29	0.2		
Intervention type	GLP-1 agonists	10	−0.35 (−3.44, 2.75)	0.82	47.6	—	0.000
	GLP-1 agonists + other treatment	2	−10.81 (−13.03, −8.59)	<0.001	14.7		

*ALT*							
Age	≥50 years old	4	−10.71 (−15.32, −6.11)	<0.001	71.1	−0.30 (−1.86, 1.26)	0.997
	<50 years old	8	−8.47 (−22.18, 5.24)	0.22	88.1		
Duration	≤12 weeks	5	−18.12 (−27.34, −8.91)	<0.001	94.2	0.53 (−0.41, 1.48)	0.003
	>12 weeks	7	−2.05 (−8.43, 4.31)	0.52	20.7		
Intervention type	GLP-1 agonists	10	−7.69 (−14.20, −1.18)	0.02	64.7	—	0.378
	GLP-1 agonists + other treatment	2	−18.40 (−41.32, 4.52)	0.11	96.6		

## Data Availability

There are no raw data associated with this review article.

## Appendix

### Search String Employed for the Systematic Review

(INDEXTERMS (“GLP-1 analog” OR “glucagon-like peptide-1 analog” OR “GLP-1 receptor agonist” OR “glucagon-like peptide-1 receptor agonist” OR “glp-1 receptor agonists” OR “glp1 receptor agonist” OR “glucagon-like” OR exenatide OR lixisenatide OR eperzan OR tanzeum OR albiglutide OR dulaglutide OR liraglutide OR semaglutide OR taspoglutide OR tanzeum OR trulicity OR byetta OR bydureon OR victoza OR adlyxin OR ozemoic OR saxenda OR bydureon OR “ITCA 650” OR “Exendin-4” OR “Exendin 4” OR byetta OR adlyxin OR lyxumia OR “rGLP-1 protein” OR ozempic) OR TITLE-ABS-KEY (“GLP-1 analog” OR “glucagon-like peptide-1 analog” OR “GLP-1 receptor agonist” OR “glucagon-like peptide-1 receptor agonist” OR “glp-1 receptor agonists” OR “glp1 receptor agonist” OR “glucagon-like” OR exenatide OR lixisenatide OR eperzan OR tanzeum OR albiglutide OR dulaglutide OR liraglutide OR semaglutide OR taspoglutide OR tanzeum OR trulicity OR byetta OR bydureon OR victoza OR adlyxin OR ozemoic OR saxenda OR bydureon OR “ITCA 650” OR “Exendin-4” OR “Exendin 4” OR byetta OR adlyxin OR lyxumia OR “rGLP-1 protein” OR ozempic)) AND (INDEXTERMS (NASH OR Liver OR “Fatty Liver” OR steatohepatitis OR “Steatosis of Liver” OR “Visceral Steatosis” OR steatosis OR “Liver Steatosis” OR “Non-alcoholic Fatty Liver Disease” OR “Non alcoholic Fatty Liver Disease” OR “NAFLD” OR “Nonalcoholic Fatty Liver Disease” OR nonalcoholic OR “Non alcoholic” OR “Nonalcoholic Fatty” OR “Non-alcoholic Fatty” OR “Nonalcoholic Fatty Liver” OR “Nonalcoholic Fatty Livers” OR “Nonalcoholic Steatohepatitis” OR steatohepatitis OR steatotic) OR TITLE-ABS-KEY (NASH OR Liver OR “Fatty Liver” OR steatohepatitis OR “Steatosis of Liver” OR “Visceral Steatosis” OR steatosis OR “Liver Steatosis” OR “Non-alcoholic Fatty Liver Disease” OR “Non alcoholic Fatty Liver Disease” OR “NAFLD” OR “Nonalcoholic Fatty Liver Disease” OR nonalcoholic OR “Non alcoholic” OR “Nonalcoholic Fatty” OR “Non-alcoholic Fatty” OR “Nonalcoholic Fatty Liver” OR “Nonalcoholic Fatty Livers” OR “Nonalcoholic Steatohepatitis” OR steatohepatitis OR steatotic)) AND (INDEXTERMS (“randomized clinical trial” OR “Randomized controlled trial” OR “random allocation” OR randomized OR “clinical trial” OR random^*∗*^ OR trial OR blind OR “controlled trial” OR “controlled study” OR “randomized trial” OR “clinical study”) OR TITLE-ABS-KEY (“randomized clinical trial” OR “Randomized controlled trial” OR “random allocation” OR randomized OR “clinical trial” OR random^*∗*^ OR trial OR blind OR “controlled trial” OR “controlled study” OR “randomized trial” OR “clinical study”)).
